# Supplementary material for: Role of Insulin Resistance as a Mediator of the Relationship between Body Weight, Waist Circumference, and Systolic Blood Pressure in a Pediatric Population
Source: Metabolites. 2023 Feb 23;13(3):327. doi: 10.3390/metabo13030327 (PMC10056524; doi:10.3390/metabo13030327)
Supplement: Supplementary file 1 [file metabolites-13-00327-s001.zip › metabolites-2144353-supplementary.pdf]

Figure S1. Mediation analysis model with DBP z-score as outcome at baseline, including HOMA-index as a mediator of the effect of BMI z-score (panel a) and WtHr (panel b) on DBP z-score.

BMI: body mass index; HOMA: Homeostasis Model Assessment; SBP: systolic blood pressure; WtHr: waist to height ratio.

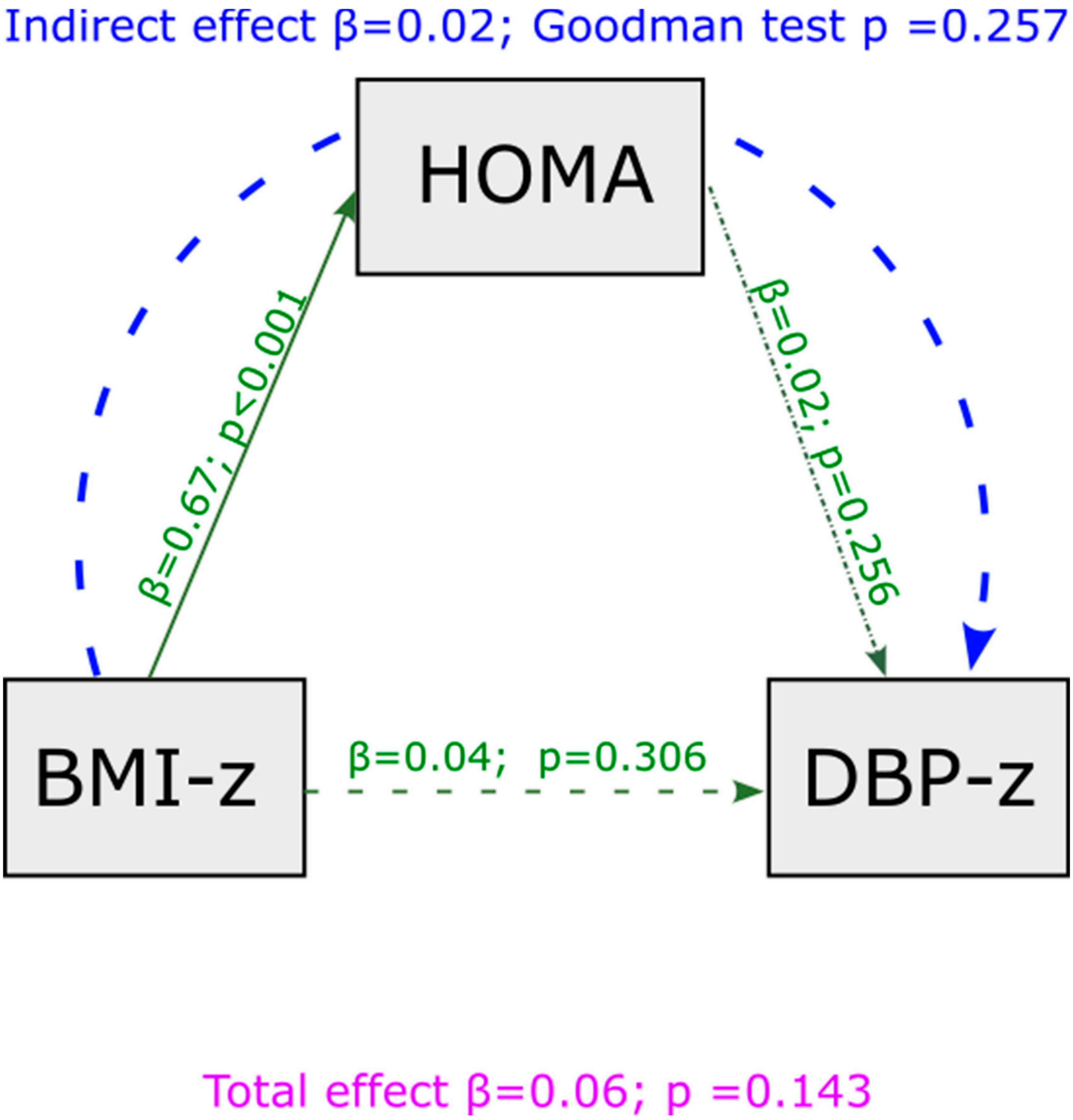

panel a

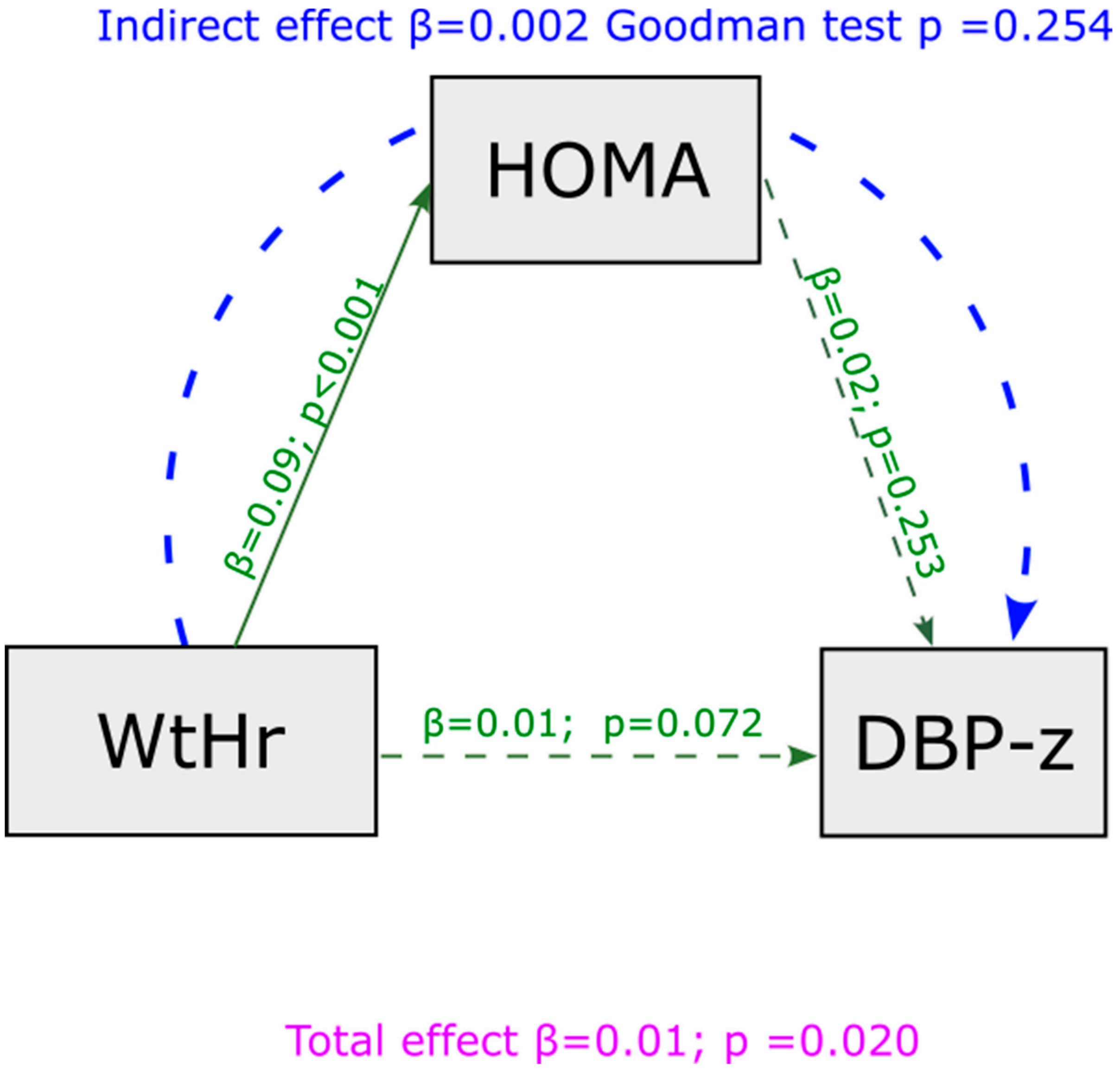
Indirect effect β=0.002 Goodman test p =0.254
HOMA
β=0.09; p<0.001
β=0.02; p=0.253
WtHr
β=0.01; p=0.072
DBP-z
Total effect β=0.01; p =0.020

panel b

Figure S2. Mediation analysis model with delta SBP z-score from baseline to follow-up as outcome, including delta HOMA-index as a mediator of the effect of delta BMI z-score (panel a) and delta WtHr (panel b) on delta SBP z-score adjusted by transition from pre-puberty to puberty.

BMI: body mass index; HOMA: Homeostasis Model Assessment; SBP: systolic blood pressure; WtHr: waist to height ratio.

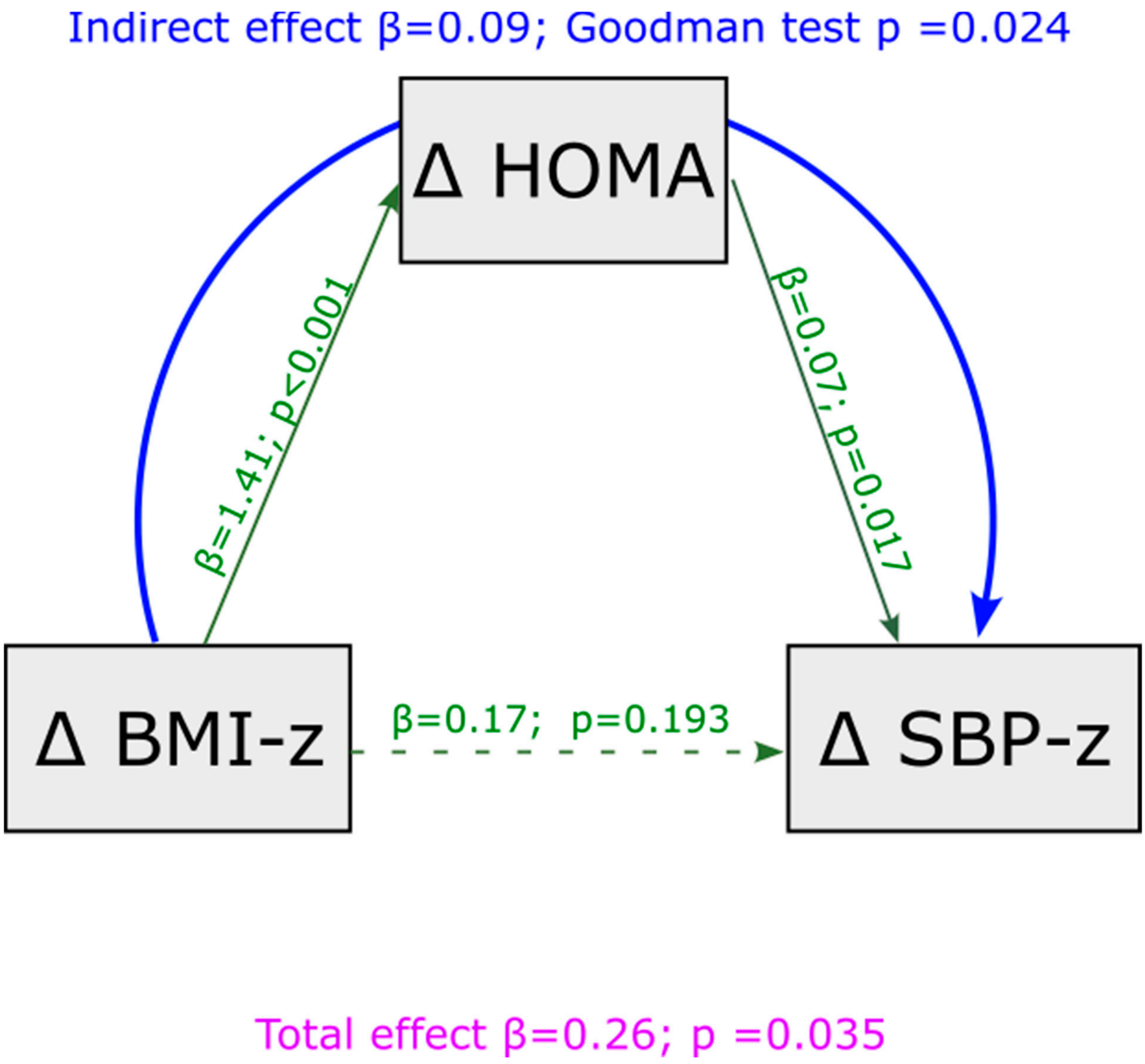

panel a

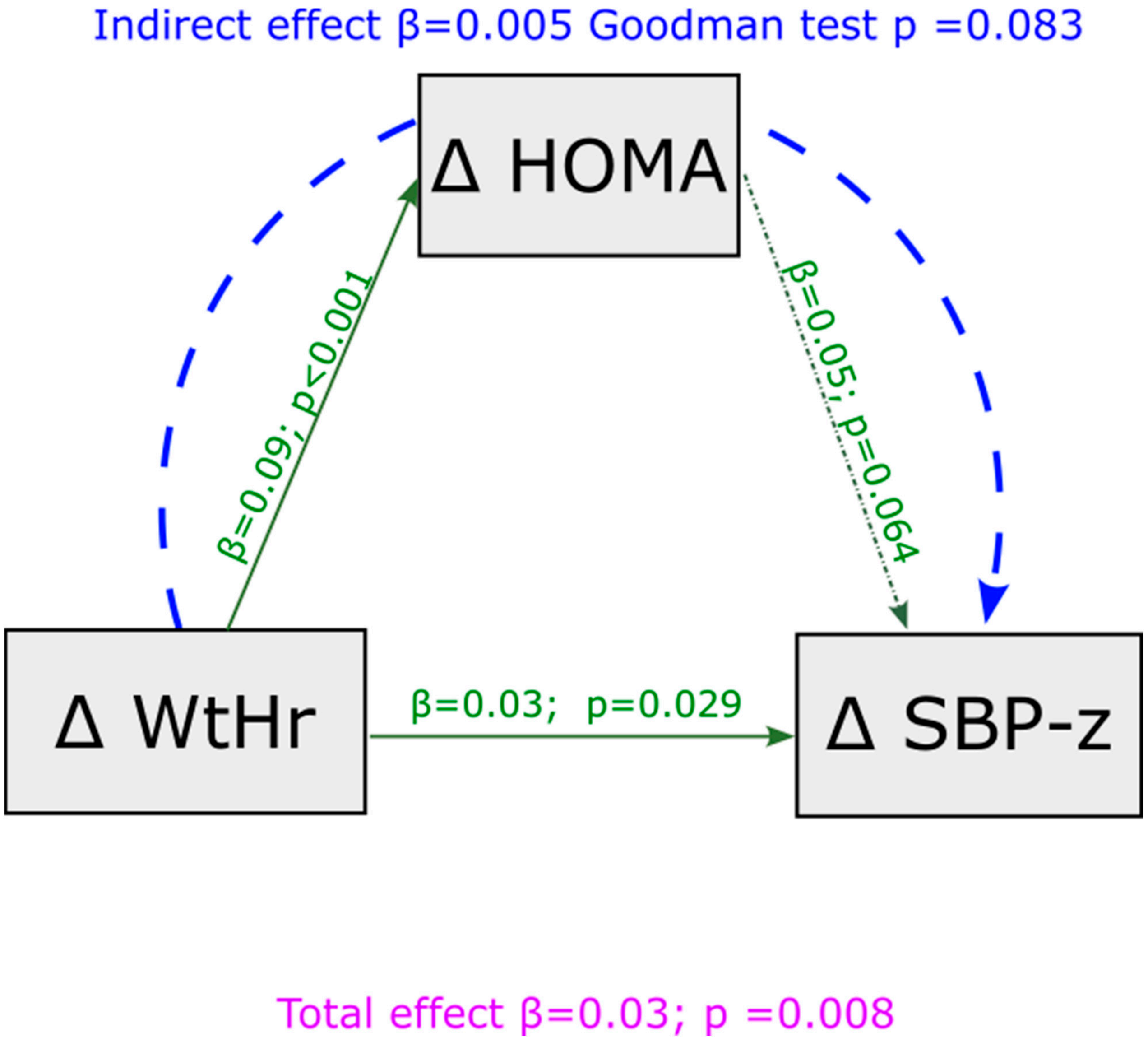

Indirect effect β=0.005 Goodman test p =0.083
Δ HOMA
β=0.09; p<0.001
β=0.05; p=0.064
Δ WtHr
β=0.03; p=0.029
Δ SBP-z
Total effect β=0.03; p =0.008

panel b

Figure S3. Mediation analysis model with delta DBP z-score from baseline to follow-up as outcome, including delta HOMA-index as a mediator of the effect of delta BMI z-score (panel a) and delta WtHr (panel b) on delta DBP z-score.

BMI: body mass index; HOMA: Homeostasis Model Assessment; SBP: systolic blood pressure; WtHr: waist to height ratio.

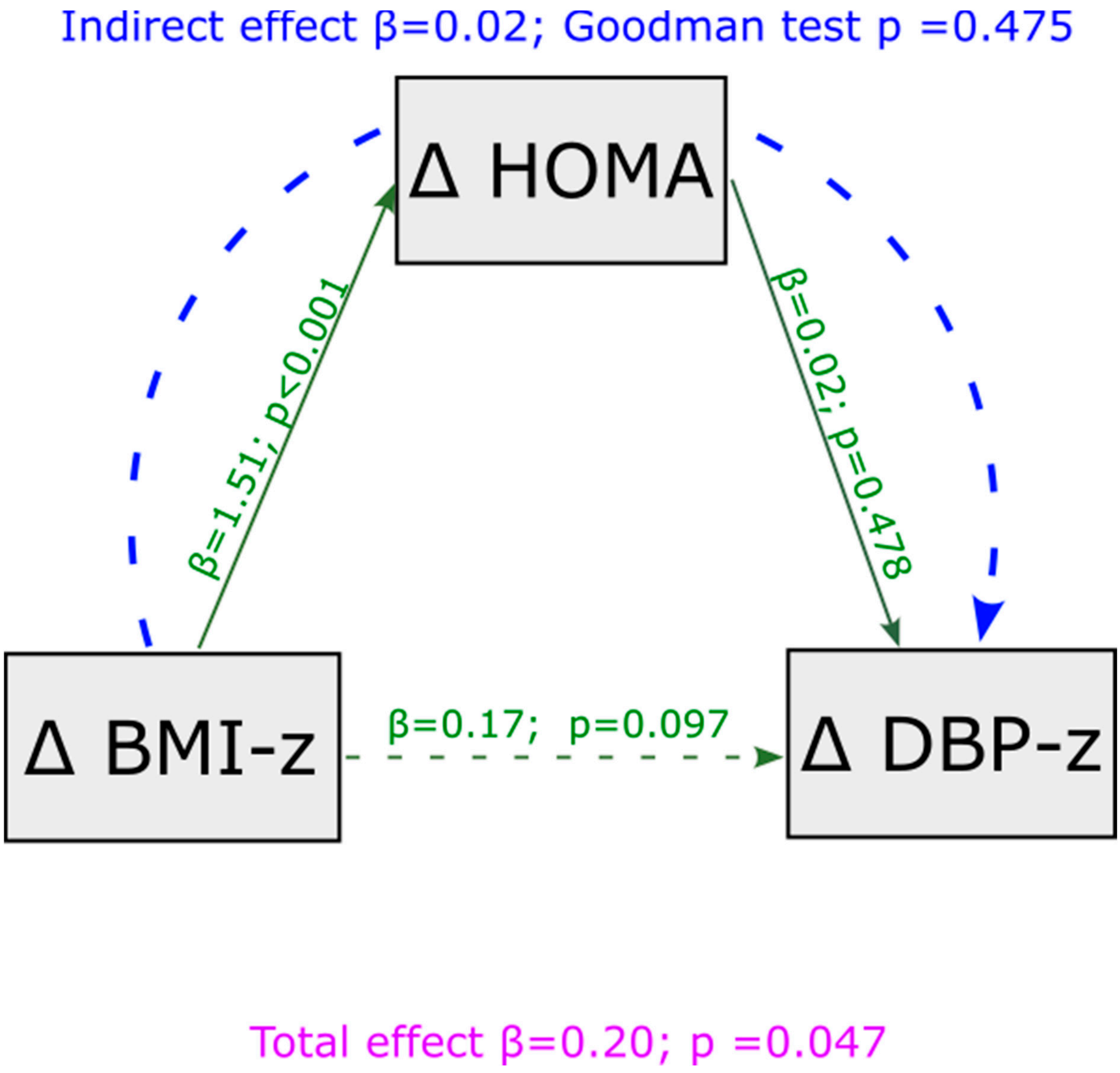

panel a

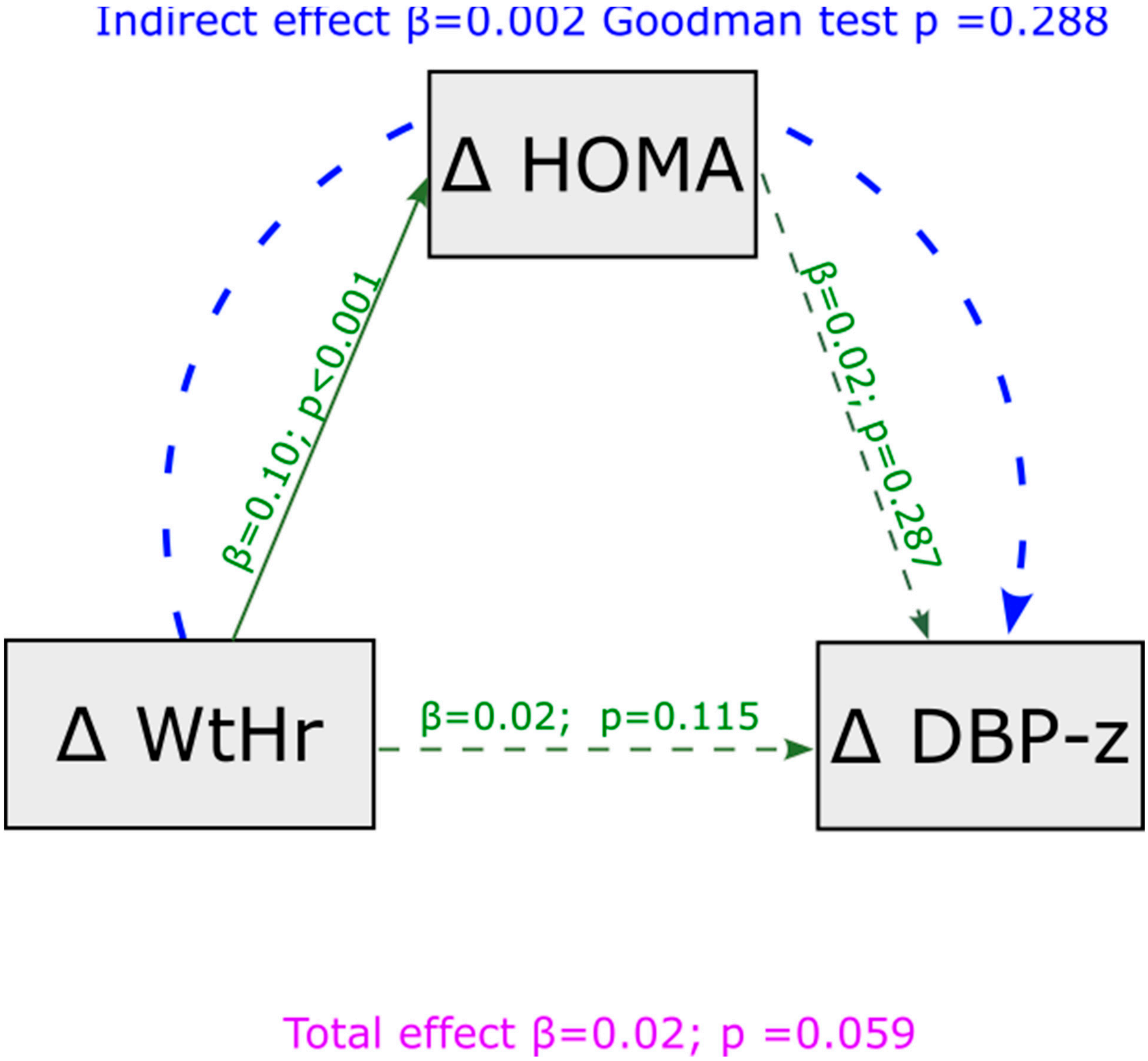
Indirect effect β=0.002 Goodman test p =0.288
Δ HOMA
β=0.10; p<0.001
β=0.02; p=0.287
Δ WtHr
β=0.02; p=0.115
Δ DBP-z
Total effect β=0.02; p =0.059

panel b
